# Myxozoan Parasite in Brain of Critically Endangered Frog

**DOI:** 10.3201/eid1804.111606

**Published:** 2012-04

**Authors:** Ashlie Hartigan, Cheryl Sangster, Karrie Rose, David N. Phalen, Jan Šlapeta

**Affiliations:** The University of Sydney, Sydney, New South Wales, Australia (A. Hartigan, D. N. Phalen, J. Šlapeta);; Taronga Conservation Society Australia, Mosman, New South Wales, Australia (C. Sangster, K. Rose)

**Keywords:** parasites, myxozoan parasites, Litoria castanea, yellow-spotted bell frog, frog disease, brain, endangered, extinction, Australia, cane toad, parasites, Australia, booroolong frog, frogs, amphibians, Cystodiscus axonis

**To the Editor:** More than three quarters of critically endangered species of amphibians are threatened by infectious disease; several are already extinct (*1*). In 2010, the yellow-spotted bell frog (*Litoria castanea*), which was presumed to be extinct, was rediscovered in the Southern Tablelands of New South Wales, Australia. This species of frog had not been seen for 30 years, and a chytrid fungus, *Batrachochytrium dendrobatidis*, was thought to be the reason (*1*,*2*). The number of frogs in the rediscovered population is estimated to be 100; if numbers are that low, the Yellow-spotted bell frog is the most critically endangered frog in Australia.

Several yellow-spotted bell frogs were collected for a captive breeding program at Taronga Zoo in Sydney, Australia. Generalized edema developed in a subadult male frog after 8 months of captivity in strict quarantine conditions. The frog subsequently died, and later an adult male frog was also found dead. Results of necropsy on both frogs at the Australian Registry of Wildlife Health revealed subcutaneous edema, intracoelomic fluid, and swollen kidneys with pale foci. Histopathologic examination demonstrated chronic severe tubulonephropathy and acute severe encephalomalacia. Coalescing foci of hemorrhage and malacia were observed in the caudal brainstem and were associated with small multinucleated (1 × 1 μm) parasites forming plasmodia-like structures 10–20 μm in diameter (Figure). Plasmodia were present in large numbers (1–5/40× field) in the spinal cord. Organisms that were morphologically consistent with myxozoan parasites detected in other frogs in Australia were found predominately within axons and were uncommonly present in vascular endothelial cells (*3*). Characteristic hepatic lesions, including lymphoplasmacytic hepatitis with biliary hyperplasia and loss of hepatocytes, were also present.

**Figure:**
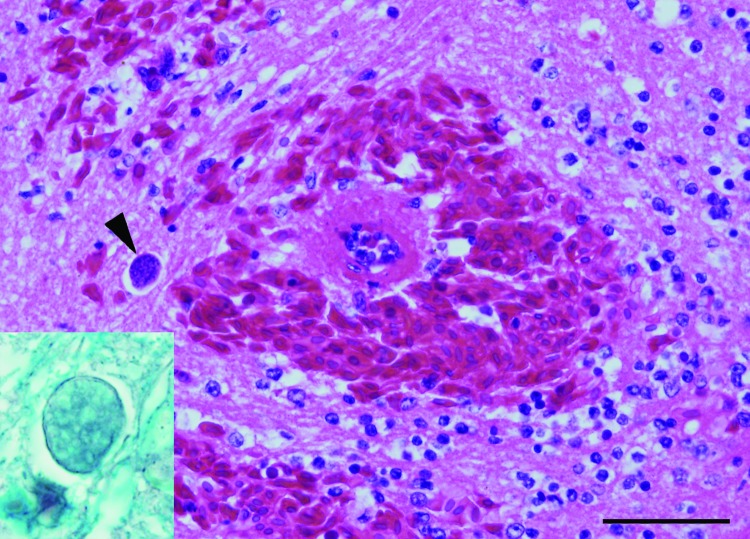
Acute severe encephalomalacia in the caudal brainstem of a captive Yellow-spotted bell frog from Sydney, Australia. This lesion was characterized by hemorrhage, vascular necrosis, and parasites consistent with Myxozoa (arrowhead) (hematoxylin and eosin stain; scale bar = 50 μm). Staining for axons confirmed intraaxonal location of the myxozoan parasites (inset, Holmes silver nitrate with Loxul Fast Blue stain).

The cause of death was renal failure, a common problem in aged frogs; however, these frogs were young, and therefore the cause of the renal changes was perplexing. We considered whether toxins (e.g., improperly cured polyvinyl chloride glue) or an infectious process might be possible causes. Staff in the zoo’s breeding program were questioned and indicated that the opportunity for introduction of a toxin was low. In addition, results for virus isolation and fungal and bacterial cultures were negative. We retrospectively reexamined histologic sections of an endangered booroolong frog (*Litoria booroolongensis*) that had similar brain lesions and intralesional myxozoan parasites (*3*). Tissue samples were submitted to the Faculty of Veterinary Science, The University of Sydney, for identification.

DNA was extracted from brain tissues (20 mg) by using the PureLink DNA Kit (Invitrogen, Mulgrave, Victoria, Australia). To test for myxozoans, we used a highly Myxozoa-specific PCR to amplify the complete internal transcribed spacer of the ribosomal DNA (*3*). Myxozoan-positive amplicons were directly sequenced at Macrogen Inc. (Seoul, South Korea), analyzed by using the CLC Main Workbench (CLC bio, Aahrus, Denmark), and deposited in GenBank (accession nos. JN977605–09).

PCR produced a 973-bp amplicon with DNA from brain and liver of the yellow-spotted bell frogs and the booroolong frog. DNA from the frogs showed 100% identity with each other, as did sequences from brain and liver. A BLASTN (*4*) search of public DNA sequence repositories returned the internal transcribed spacer of the ribosomal DNA of a myxozoan parasite, *Cystodiscus axonis* (syn. *Myxidium* sp. ‘brain’), as the most closely related sequence (*3*,*5*). Pairwise comparison revealed 100% identity with *C. axonis*. During the brain phase of infection, *C. axonis* parasites reside within the myelinated axons (*5*), and special staining for axons confirmed this location for the brain parasites in this study. Screening of tissue sections from frogs trapped in the same locality as the yellow-spotted bell frogs revealed the presence of the parasite in the central nervous system of 8 of 10 stony creek frogs (*Litoria wilcoxi*) and 1 of 5 eastern banjo frogs (*Limnodynastes dumerilii*).

Little information exists about the pathologic significance of myxozoan parasite in frogs and tadpoles (*6*). In Australia, *Cystodiscus* spp. parasites of frogs are emerging and have spread widely along the eastern coast in the past 40 years; they were first detected in a frog collected in 1966 (*7*). Molecular characterization revealed 2 cryptic *Cystodiscus* parasites in frogs endemic to Australia and in the invasive cane toad (*3*). However, the cane toad did not introduce this parasite into Australia because cane toads from Hawaii, which are devoid of the parasite, were the source population for toads in Australia. The parasites seem to be native to Australia, and the invasive cane toad plays a spill-back role in their dissemination; however, it is not known how these parasites were disseminated outside the cane toad range (*3*).

Frog myxozoan parasites are yet to be documented as a cause of population decline; yet, the frequent presence of these parasites in moribund animals in captivity, including the yellow-spotted bell frog, demonstrates the need to monitor parasites in endangered frog populations worldwide. On the basis of our necropsy findings in the central nervous system of 2 yellow-spotted bell frogs, we encourage other investigators to consider the potential role that myxozoan parasites may play in wild and captive populations of declining frogs worldwide.
